# Supplementary material for: ZHX2 emerges as a negative regulator of mitochondrial oxidative phosphorylation during acute liver injury
Source: Nat Commun. 2023 Nov 18;14:7527. doi: 10.1038/s41467-023-43439-0 (PMC10657347; doi:10.1038/s41467-023-43439-0)
Supplement: Supplementary file 1 — Supplementary Information [file 41467_2023_43439_MOESM1_ESM.pdf]

Supplemental information

ZHX2 emerges as a negative regulator of mitochondrial oxidative phosphorylation during acute liver injury

This file includes:

Supplementary Figures 1 to 7

Supplementary Tables 1 to 2

Supplementary Figures and Figure legends

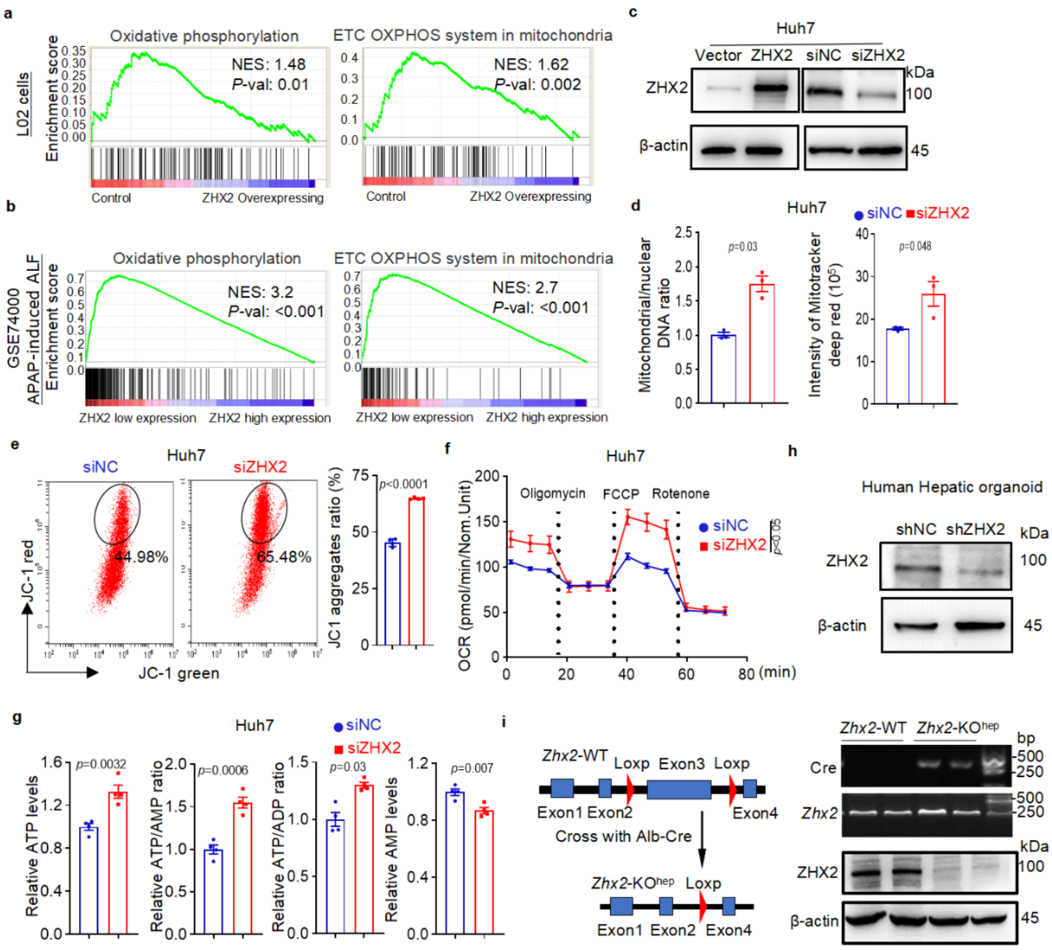

Supplementary Figure 1. ZHX2 inhibits mitochondrial OXPHOS *in vitro* and *in vivo*.

a-b GSEA analysis were performed with publicly available data, including ZHX2-overexpressed LO2 cells, APAP-induced acute liver failure (ALF) (GSE74000), respectively. NES, normalized enrichment score, P<0.05 was considered statistically significant. Data were analyzed using Kolmogorov-Smirnov test. **c** Western blot analysis for ZHX2 in Huh7 cells with ZHX2 ectopic expression or knockdown. These experiments have been repeated for three times with similar results. **d** Detection of copy number of mtDNA (left) and intensity of Mito tracker deep red (right) in Huh7 cells with or without ZHX2 knockdown, respectively. Representative data are presented as mean ± sd. (Two-tailed Student' s t test.

n=3 biologically independent samples). **e** Fluorescence intensity of JC-1 in Huh7 cells with or without ZHX2 knockdown were analyzed by flow cytometry. Representative flow cytometry plots are presented in the left panel. Histograms display the quantitative data in the right panel. Representative data are presented as mean ± sd. (Two-tailed Student' s t test. n=4 biologically independent samples). **f** Relative oxygen consumption rate was measured siNC and siZHX2 transfected Huh7 cells by seahorse analyzer. The data were normalized to protein abundance. Representative data are presented as mean ± sd. (Two-tailed Student' s t test. n=3 biologically independent samples). **g** Levels of ATP and AMP, and ratios of ATP/AMP and ATP/ADP were determined in Huh7 cells transfected with siNC and siZHX2. Representative data are presented as mean ± sd. (Two-tailed Student' s t test. n=4 biologically independent samples). **h** Western blot analysis for knockdown efficiency of ZHX2 in human hepatic organoid. These experiments have been repeated for three times with similar results. **i** Establishment of liver-specific ZHX2 knockout mice were confirmed. Schematic diagram of *Zhx2*-KO^{hep}(left) is presented (Left). Mouse tails were collected to extract DNA for genotyping by PCR (Top right). ZHX2 protein levels were detected in *Zhx2*-WT and *Zhx2*-KO^{hep} mice liver tissues by western blot (Bottom right).

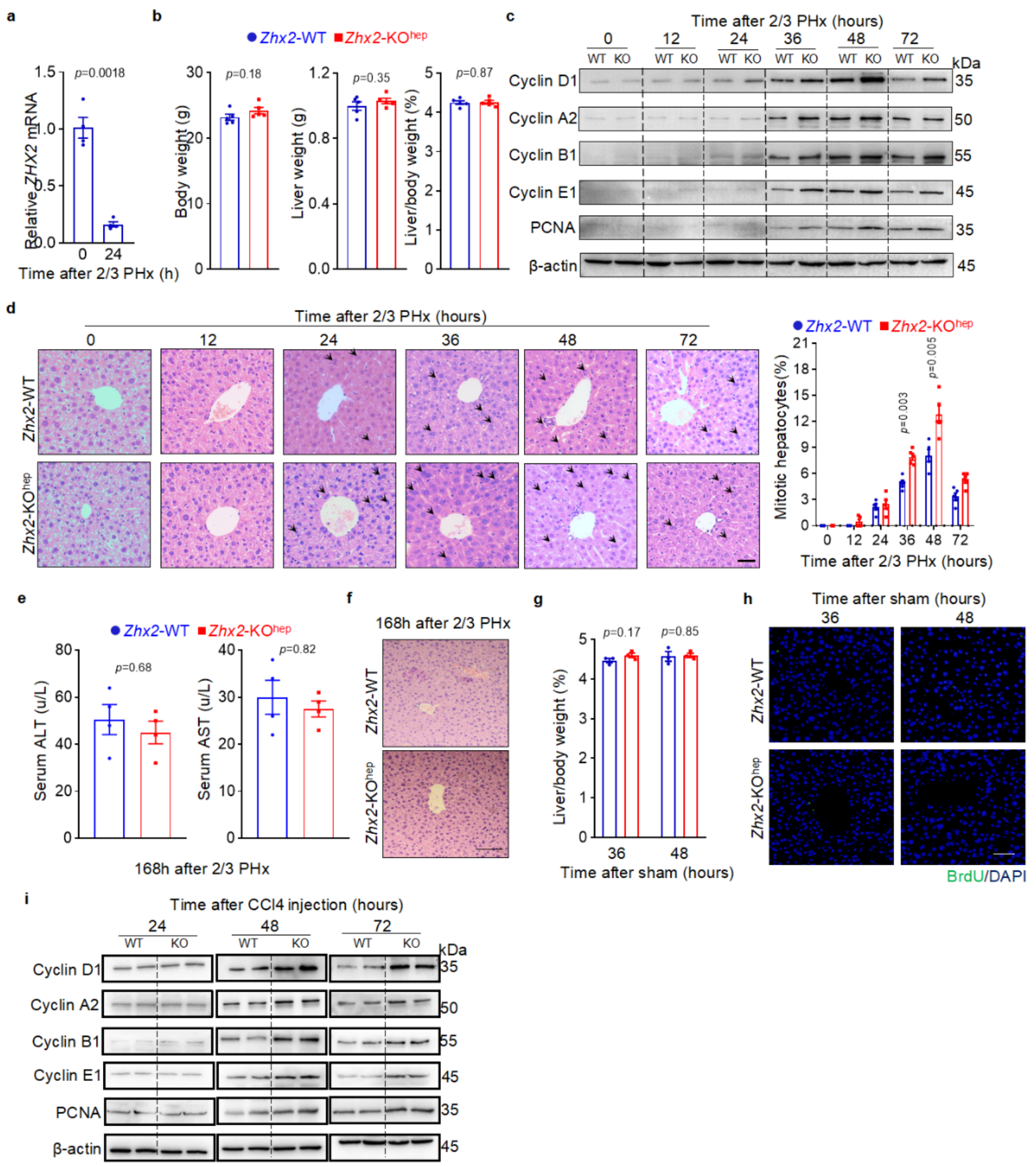

Supplementary Figure 2. Hepatic *Zhx2* deficiency enhances liver repair in 2/3 PHx and CCl4-indcued liver injury.

a ZHX2 expression was determined in wild type mouse liver at 0 and 24 h after 2/3 PHx and by RT-qPCR. Data are presented as mean ± s.e.m. (Two-tailed Student' s t test. n=4 mice per group). **b** Liver weight, body weight, and the liver/body weight ratios of 8-10-week-old *Zhx2*-WT and *Zhx2*-KO^{hep} mice were determined. Data are presented as mean ± s.e.m. (Two-tailed Student' s t test. n=5 mice). **c** The protein levels of Cyclin D1, Cyclin A2, Cyclin B1, Cyclin E1 and PCNA were detected in *Zhx2*-WT and *Zhx2*-KO^{hep} mice at indicated time

points after 2/3 PHx by western blot (WT: *Zhx2*-WT, KO: *Zhx2*-KO^{hep}). These experiments have been repeated for three times with similar results. **d** Representative images of mitotic hepatocytes (indicated by arrows) of liver sections from *Zhx2*-WT and *Zhx2*-KO^{hep} mice at indicated time points after 2/3 PHx are displayed at left panel. The quantitative data are presented on right panel. Scale bar: 50 μm. Data are represented as mean ± s.e.m. (Two-tailed Student' s t test. n=5 mice per group). **e-f** The ALT, AST (e) and H&E (f) were determined in *Zhx2*-WT and *Zhx2*-KO^{hep} mice at 168 h after 2/3 PHx. Scale bar: 100 μm. Data are presented as mean ± s.e.m. (Two-tailed Student' s t test. n=4 mice per group). **g-h** The liver/body weight ratios (g) and BrdU (h) were determined in *Zhx2*-WT and *Zhx2*-KO^{hep} mice at 36 h and 48 h after sham operation. Scale bar: 50 μm. Data are presented as mean ± s.e.m. (Two-tailed Student' s t test. n=3 mice per group). **i** The protein levels of proliferation-related genes, such as CyclinD1, CyclinA2, CyclinB1, Cyclin E1 and PCNA, were detected in the livers of *Zhx2*-WT and *Zhx2*-KO^{hep} mice after CCl4 injection, respectively. These experiments have been repeated for three times with similar results.

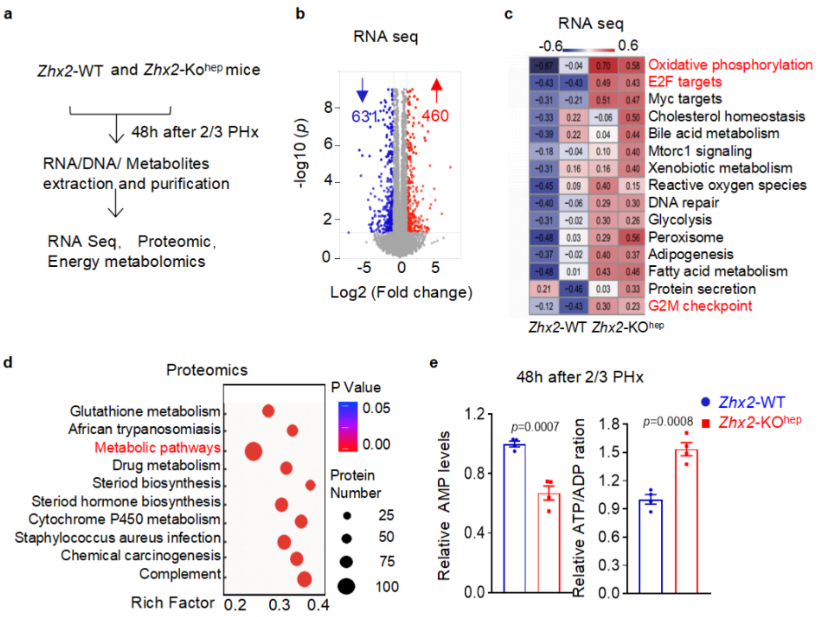

Supplementary Figure 3. Multi-omics analysis reveals enhanced OXPHOS activity in hepatocytes with *Zhx2* deficiency during liver repair.

a Schematic of the experimental strategy used to identify the potential target of ZHX2 in the samples of *Zhx2*-WT and *Zhx2*-KO^{hep} mice after 2/3 PHx. **b** Volcano plot represented differentially expressed genes between *Zhx2*-WT and *Zhx2*-KO^{hep} mice liver tissues. The significantly up-regulated genes were labeled as red, and the significantly down-regulated genes were labeled as blue. **c** RNA-seq was performed with liver tissues from *Zhx2*-KO^{hep} and *Zhx2*-WT mice at 48 h after 2/3 PHx. GSVA were used to analyze the genes with differential mRNA levels. **d** KEGG analysis of the differential proteins from *Zhx2*-WT and *Zhx2*-KO^{hep} mice showed top enriched categories. **e** AMP levels and ATP/ADP ratio in hepatocytes from *Zhx2*-KO^{hep} and *Zhx2*-WT mice were accessed. Data are presented as mean ± s.e.m. (Two-tailed Student' s t test. n=4 mice per group).

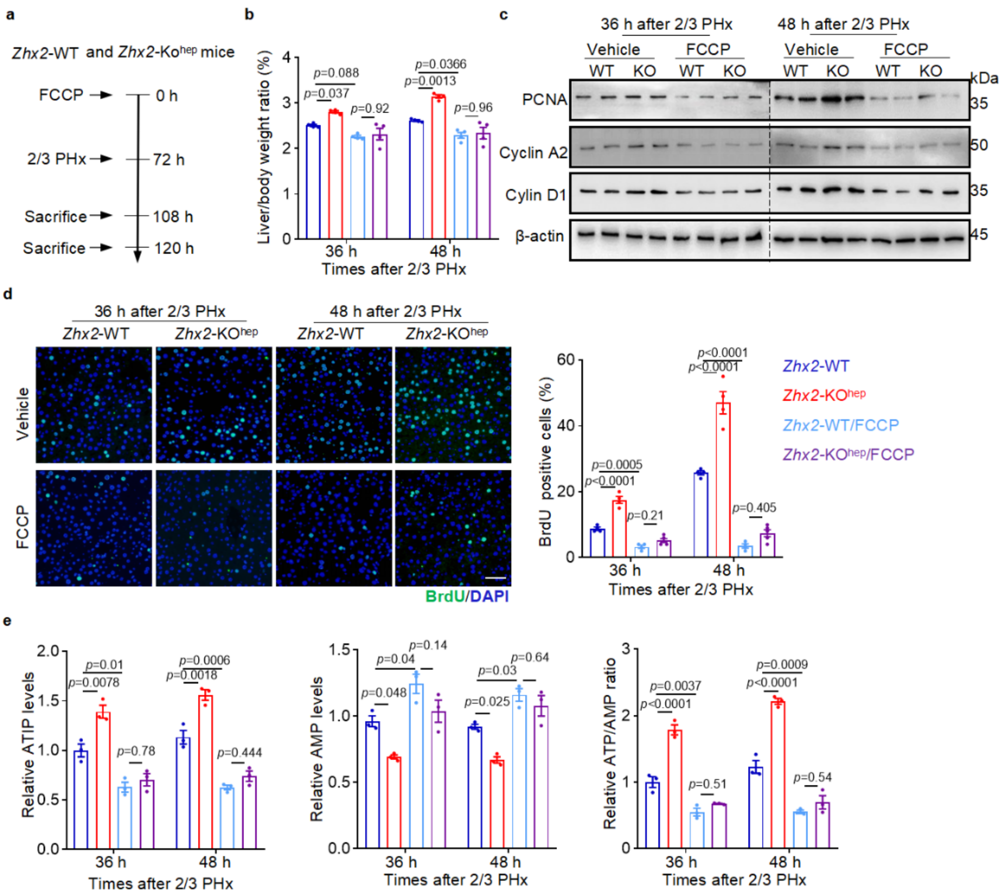

Supplementary Figure 4. FCCP treatment abolishes *Zhx2* deletion-induced augmentation of mitochondrial OXPHOS and liver recovery.

a Diagram of FCCP administration in *Zhx2*-KO^{hep} and *Zhx2*-WT mice with 2/3 PHx was presented. **b** Liver/body weight ratios of *Zhx2*-WT and *Zhx2*-KO^{hep} mice with or without FCCP administration were measured at 36 h and 48 h after 2/3 PHx, respectively. Data are presented as mean ± s.e.m. (One-way ANOVA with Tukey's test. n=4 mice per group). **c** Expression of PCNA, Cyclin A2 and Cyclin D1 in *Zhx2*-WT and *Zhx2*-KO^{hep} livers with or without FCCP treatment were determined at 36 h and 48 h after 2/3 PHx by western blot. These experiments have been repeated for three times with similar results. **d** BrdU-positive cells in livers from FCCP and vehicle treated *Zhx2*-KO^{hep} and *Zhx2*-WT mice were determined, respectively. Representative images (left) and quantitative data (right) were

presented. Scale bar: 50 μm. Data are presented as mean ± s.e.m. (One-way ANOVA with Tukey's test. n=4 mice per group). **e** ATP and AMP levels, and ATP/AMP ratio of FCCP and vehicle administrated *Zhx2*-KO^{hep} and *Zhx2*-WT livers were determined at 36 h and 48 h after 2/3 PHx. Data are presented as mean ± s.e.m. (One-way ANOVA with Tukey's test. n=3 mice per group).

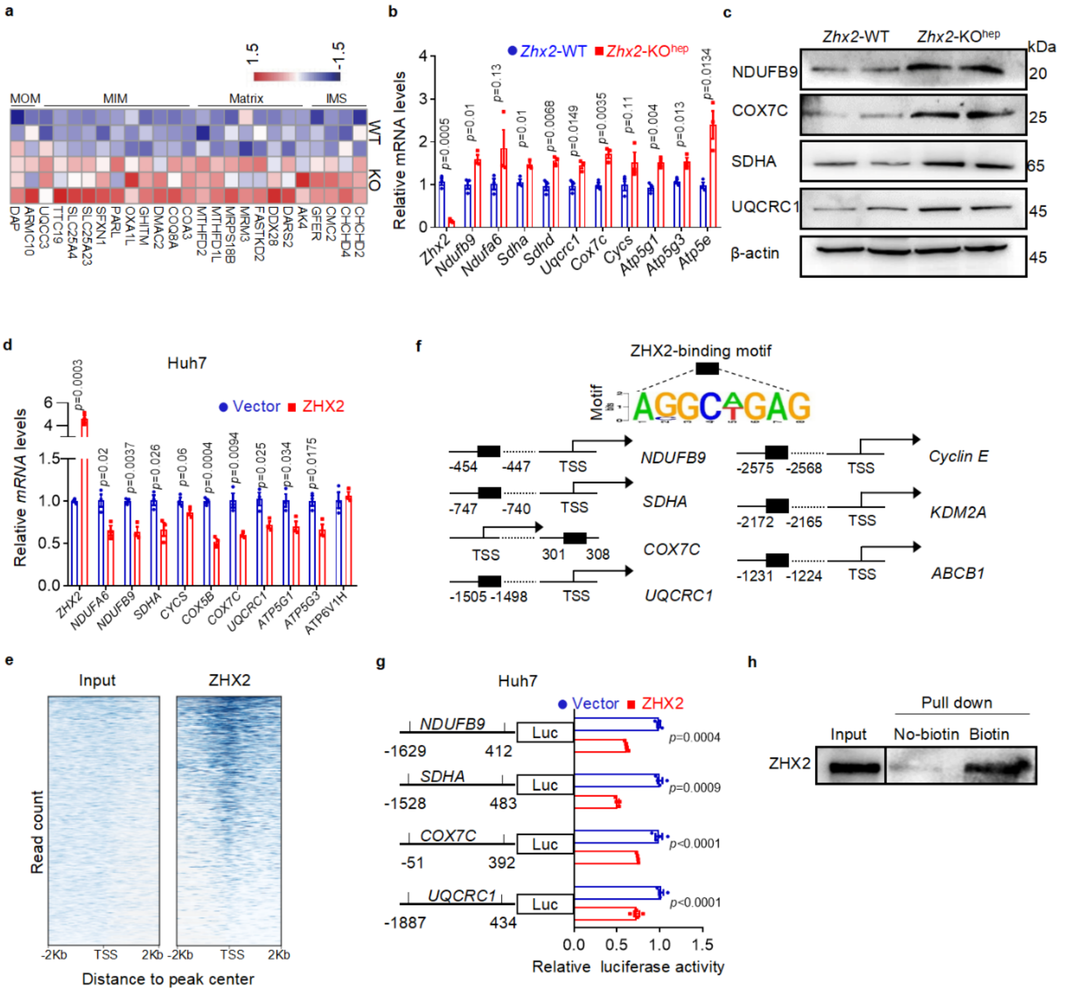

Supplementary Figure 5. ZHX2-binding motif is located on the promoter of ETC genes.

a Differentially expressed mitochondrial related proteins detected by the proteomics were presented as heatmap. MOM, mitochondrial outer membrane; MIN, mitochondrial inner membrane; IMS, intermembrane space. **b** Expression of ETC genes were assessed in hepatocytes of *Zhx2*-KO^{hep} and *Zhx2*-WT mice at 48 h after 2/3 PHx by RT-qPCR. Data are presented as mean ± s.e.m. (Two-tailed Student' s t test. n=3 mice per group). **c** The protein levels of ETC components were assessed in hepatocytes from *Zhx2*-KO^{hep} and *Zhx2*-WT mice at 48 h after 2/3 PHx by western blot. These experiments have been

repeated for three times with similar results. **d** Relative mRNA levels of ETC genes were measured in Huh7 cells with ZHX2 overexpression by RT-qPCR. Representative data are presented as mean ± sd. (Two-tailed Student’ s t test. n=3 biologically independent samples). **e** Heatmap displayed ChIP-Seq signal density for ZHX2 ChIP and input centered on predicted Transcription Start Site (TSS). **f** The diagram shows the sequence of ZHX2-binding putative motif and the location of this motif on the promoter of ZHX2 target genes. **g** The promoter activity of indicated ETC genes were determined in Huh7 cells with or without ZHX2 overexpression by dual-luciferase assays. The schematic representation of the promoter regions for ETC genes were displayed on the left. Representative data are presented as mean ± sd. (Two-tailed Student’ s t test. n=4 biologically independent samples). **h** Biotin-labeled ZHX2-binding motif (probe) was incubated with nuclear protein of Huh7 cells with ZHX2 overexpression to validate the binding of ZHX2 with the consensus motif.

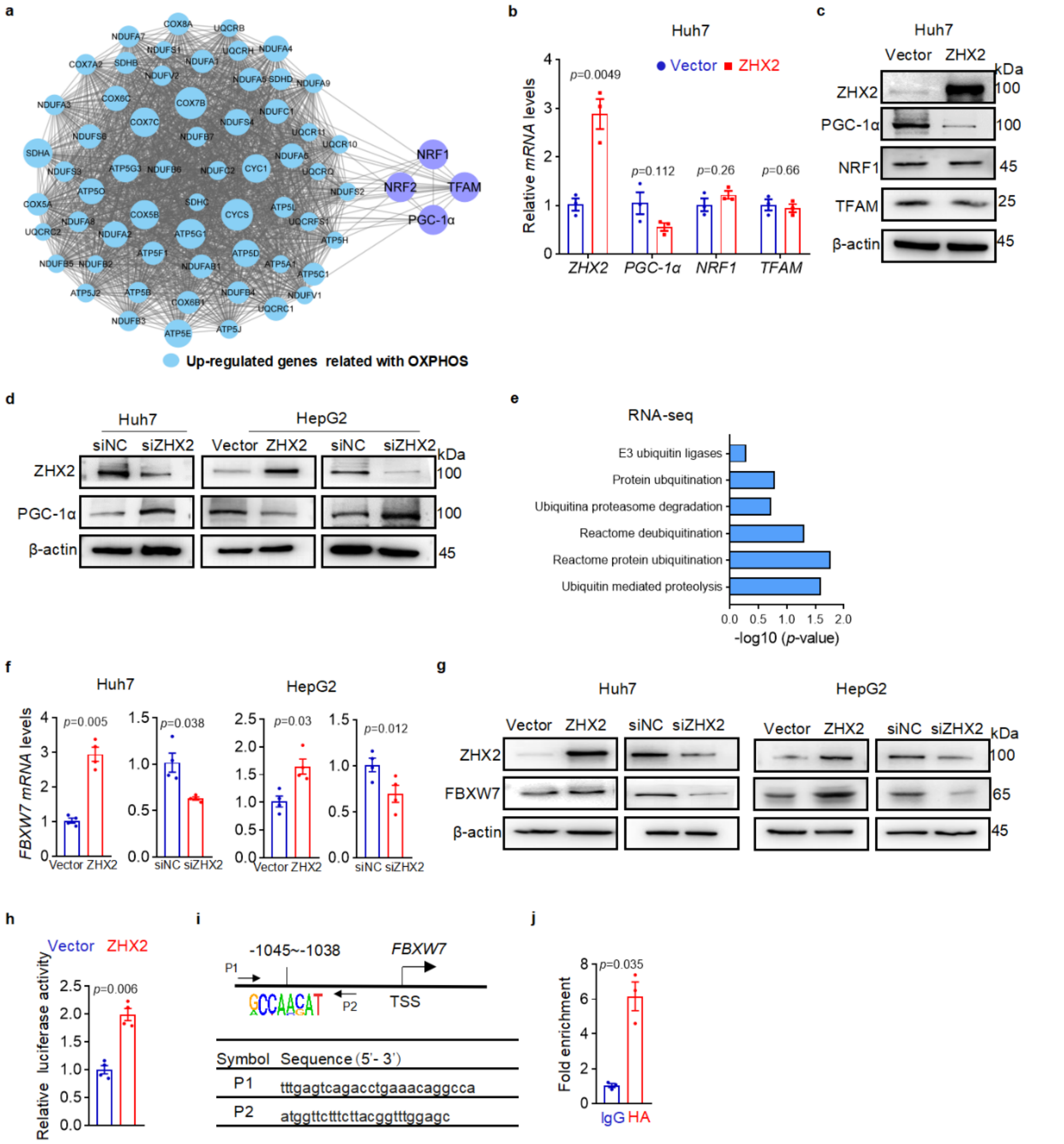

Supplementary Figure 6. ZHX2 decreases PGC-1α protein levels through up-regulation of FBXW7.

a The relationship of mitochondrial regulators (PGC-1α, NRF1/2 and TFAM) with ZHX2-regulated mitochondrial OXPHOS genes were analyzed by co-expression network. **b** The mRNA levels of *PGC-1α*, *NRF1* and *TFAM* were detected in ZHX2 and control vectors transfected Huh7 cells by RT-qPCR. Representative data are presented as mean ± sd. (Two-tailed Student’ s t test. n=3 biologically independent samples). **c** The protein levels

of PGC-1α, NRF1and TFAM in Huh7 cells transfected with ZHX2 and control vector were detected by western blot. These experiments have been repeated for three times with similar results. **d** The expression of PGC-1α were detected in Huh7 and HepG2 cells with ZHX2 manipulation by western blot. **e** GSEA analysis showed the enrichment of differentially expressed ubiquitination related gene sets in *Zhx2*-KO^{hep} mice at 48 h after 2/3 PHx. **f** Relative mRNA levels of FBXW7 in Huh7 and HepG2 cells with ZHX2 overexpression or knockdown were measured by RT-qPCR, respectively. Representative data are presented as mean ± sd. (Two-tailed Student’ s t test. n=4 biologically independent samples). **g** The expression of FBXW7 were detected in ZHX2-manipulated Huh7 and HepG2 cells by western blot. These experiments have been repeated for three times with similar results. **h** The luciferase reporter vector containing FBXW7 promoter was co-transfected with ZHX2-HA or control vectors in Huh7 cells. The promoter activities were displayed as the luciferase intensity. Representative data are presented as mean ± sd. (Two-tailed Student’ s t test. n=4 biologically independent samples). **i** The upper panel showed location of ZHX2-binding motif on FBXW7 promoter detected in the ChIP-Seq data. The bottom panel showed primer sequences for ChIP assays. **j** ChIP assay was performed with anti-HA antibody, IgG as control, using Huh7 cells transfected with ZHX2-HA. ZHX2 occupied at the promoter of FBXW7 was quantified by qPCR. Representative data are presented as mean ± sd. (Two-tailed Student’ s t test. n=3 biologically independent samples).

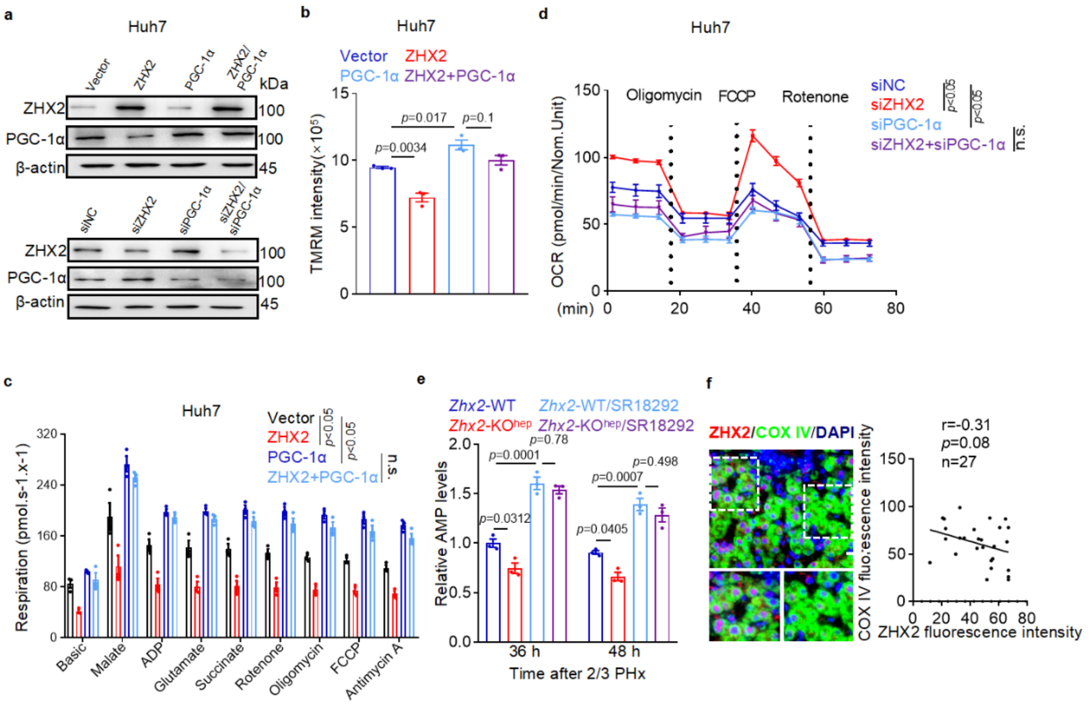

Supplementary Figure 7. Loss of *Zhx2* enhances mitochondrial OXPHOS by stabilization of PGC-1α.

a The protein levels of PGC-1α and ZHX2 were detected in Huh7 cells with ZHX2/PGC-1α overexpression or knockdown by western blot, respectively. These experiments have been repeated for three times with similar results. **b** The fluorescence intensity of TMRM were analyzed in Huh7 cells with indicated manipulation by flow cytometry. The quantitative data were presented. Representative data are presented as means ± sd. (One-way ANOVA with Tukey's test. n=3 biologically independent samples). **c** Oxygen consumption was analyzed in Huh7 cells with indicated manipulation by oxygraph-2k assay kit. Representative data are presented as means ± sd. (One-way ANOVA with Tukey's test. n=3 biologically independent samples. n.s. indicates the difference is not significant). **d** Oxygen consumption was analyzed in Huh7 cells with indicated manipulation by using seahorse analyzer. Representative data are presented as means ± sd. (Two-tailed Student'

s t test. n=4 biologically independent samples. n.s. indicates the difference is not significant). **e** AMP levels of vehicle or 18292 treated *Zhx2*-WT and *Zhx2*-KO^{hep} mice liver were determined at 36 h and 48 h after 2/3 PHx. Data are presented as mean ± s.e.m. (*One-way ANOVA with Tukey's test. n=3 mice per group). **f** Correlation analysis of fluorescence intensity of ZHX2 and COX IV in DILI patients. Right, representative images. Left, quantitative data. Scale bar: 20 μm. Pearson's correlation coefficients (r) and *p* values (*p*) for two-sided correlation tests are shown.

Supplementary tables

Supplementary Table 1 Characteristics of DILI patients

	**All patients**	**Hepatocellular injury**	**Cholestatic injury**	**Mixed injury**
**n**	**27**	**17**	**3**	**7**
Age (years)	48.48±12.83	46.88±14.20	50.00±17.44	51.71±7.34
Gender (F/M)	16/11	9/8	2/1	5/2
ALT (U/L)	228.45±284.83	293.95±339.53	44.60±49.38	148.16±82.12
AST (U/L)	224.59±269.27	269.44±312.51	63.30±7.18	184.80±182.1
GGT (U/L)	265.59±303.01	285.10±331.16	276.73±407.80	213.43±213.6
ALP (U/L)	154.29±97.82	162.34±110.82	139.50±81.67	141.07±77.44
TBIL	104.09±131.29	72.88±96.71	160.77±173.05	155.59±179.7
TBA	91.03±102.12	88.66±85.42	100.73±96.29	92.64±150.26
Severity, n				
Mild/Moderate	13	7	3	3
Severe/Fatal	14	10	0	4

ALP alkaline phosphatase, ALT alanine aminotransferase, AST aspartate aminotransferase, GGT gamma glutamyl transferase, TBIL total bilirubin, TBA total bile acid. Data are expressed as mean ± SD.

Supplementary Table 2

Synthetic Oligonucleotides.

Primer for RT-qPCR

**Symbol**	**Sequence (5'- 3')**
h-ZHX2-F	GGTTCGGACATCACAAGTAGTAG
h-ZHX2-R	GGTGTGCCGATTCCTTTCTCT
h-β-actin-F	AGTTGCGTTACACCCTTTC
h-β-actin-R	CCTTCACCGTTCCAGTTT
h-NDUFB9-F	CTGGGAACGAGAGGTTAAGCA
h-NDUFB9-R	GGGTCTGGTCACAATATACCACC
h-SDHA-F	CAGCATGTGTTACCAAGCTGT
h-SDHA-R	GGTGTCGTAGAAATGCCACCT
h-UQCRC1-F	GGGGCACAAGTGCTATTGC
h-UQCRC1-R	GTTGTCCAGCAGGCTAACC
h-COX7C-F	GGTCCGTAGGAGCCACTATGA
h-COX7C-R	GTGTCTTACTACAAGGAAGGGTG
h-ATP6V1H-F	GCAAAGAACAGACCGTTCAGT
h-ATP6V1H-R	ATTGGCAGAAAGTAGGGCCAC
h-NDUFA6-F	CGCCAAGCTACTTCTACCGC
h-NDUFA6-R	TCGGACTTTATCCCGTCCCA
h-CYCS-F	CTTTGGGCGGAAGACAGGTC
h-CYCS-R	TTATTGGCGGCTGTGTAAGAG
h-COX5B-F	TGTGAAGAGGACAATACCAGCG
h-COX5B-R	CCAGCTTGTAATGGGCTCCAC
h-ATP5G1-F	TTCCAGACCAGTGTTGTCTCC
h-ATP5G1-R	GACGGGTTCCTGGCATAGC
h-ATP5G3-F	CCAGAGTTGCATACAGACCAAT
h-ATP5G3-R	CCCATTAAATACCGTAGAGCCCT
h-PGC-1α-F	CCAAAGGATGCGCTCTCGTTCA

h-PGC-1α-R	CGGTGTCTGTAGTGGCTTGACT
h-NRF1-F	AGGAACACGGAGTGACCCAA
h-NRF1-R	TATGCTCGGTGTAAGTAGCCA
h-TFAM-F	GTGGTTTTCATCTGTCTTGGCAAG
h-TFAM-R	TTCCCTCCAACGCTGGGCAATT
h-ND1-F	CCCTAAAACCCGCCACATCT
h-ND1-R	GAGCGATGGTGAGAGCTAAGGT
h-B2M-F	CCAGCAGAGAATGGAAAGTCAA
h-B2M-R	TCTCTCTCCATTCTTCAGTAAGTCAACT
h-FBXW7-F	GGCGCCGCGGCTCTTTTCTA
h-FBXW7-R	GCTGCCCACAGAGAGCAGTTCC
h-RNF34-F	GGAGAGCTTATGGATGGAGACC
h-RNF34-R	GGTTCCGATCCTCTGCGTT
m-ZHX2-F	TGGAAGCGAGGCGGCACATCAG
m-ZHX2-R	CCGGCTCCAGCTACCCCACTTCTC
m-β-actin -F	TGCGTGACATCAAAGAGAAG
m-β-actin -R	TCCATACCCAAGAAGGAAGG
m-Cyclin A2-F	ACAGAGTGTGAAGATGCCCTGGCT
m-Cyclin A2-R	AGCATGTGGTGATTCAAAACTGCCA
m-Cyclin B1-F	AAGGTGCCTGTGTGTGAACC
m-Cyclin B1-R	GTCAGCCCCATCATCTGCG
m-Cyclin D1-F	GCGTACCCTGACACCAATCTC
m-Cyclin D1-R	CTCCTCTTCGCACTTCTGCTC
m-Cyclin E1-F	GTGGCTCCGACCTTTCAGTC
m-Cyclin E1-R	CACAGTCTTGTCAATCTTGGCA
m-NDUFB9-F	AAGGTGCTGCGGCTGTATAAG
m-NDUFB9-R	TCATCAAGCAAGCAAAGTACCG
m-SDHA-F	GGAACACTCCAAAAACAGACCT
m-SDHA-R	CCACCACTGGGTATTGAGTAGAA
m-SDHD-F	TGGTCAGACCCGCTTATGTG

m-SDHD-R	GAGCAGGGATTCAAGTACCCA
m-UQCRC1-F	AGACCCAGGTCAGCATCTTG
m-UQCRC1-R	GCCGATTCTTTGTTCCCTTGA
m-COX7C-F	ATGTTGGGCCAGAGTATCCG
m-COX7C-R	ACCCAGATCCAAAGTACACGG
m-ATP5G1-F	TTCTCCAGCTCTGATTCGCTC
m-ATP5G1-R	CCGGGAAATGACACTGGTCT
m-ATP5G3-F	CTGGTATTGGAACAGTCTTTGGC
m-ATP5G3-R	GATCAAGAACGCAACCATCAAAC
m-ATP5B-F	TCCTGCCAGAGACTATGCG
m-ATP5B-R	GATGACTGCCACGATTCGC
m-D-loop-F	AATCTACCATCCTCCGTGAAACC
m-D-loop-R	TCAGTTTAGCTACCCCCAAGTTTAA
m-B2M-F	ATGGGAAGCCGAACATACTG
m-B2M-R	CAGTCTCAGTGGGGGTGAAT
m-NDUFA6-F	TCGGTGAAGCCCATTTTCAGT
m-NDUFA6-R	CTCGGACTTTATCCCGTCCTT
m-CYCS-F	CCAAATCTCCACGGTCTGTTC
m-CYCS-R	ATCAGGGTATCCTCTCCCCAG
m-FBXW7-F	GTTCCGCTGCCTAATCTTCCT
m-FBXW7-R	CCCTTCAGGGATTCTGTGCC
m-RNF34-F	GAAACATACCAACCGACACTTGT
m-RNF34-R	AGGCTACTTGAGTCCAGGTCA

Primer for ChIP-PCR

**Symbol**	**Sequence (5'- 3')**
NDFB9-ChIP-F	CTCCACCAGATGGTGATGAC
NDFB9-ChIP-R	ATTCATCCCACGTGCACCT
SDHA-ChIP-F	CACCGGACACTTTCATATGAGCTAGG
SDHA-ChIP-R	GTTTGCACCTTCCCCACATCAC
COX7C-ChIP-F	CTGTGACTCGCGCACCT

COX7C-ChIP-R	CGCTAGGATGCCCAGCT
UQCRC1-ChIP-F	AAATAAAGTCAGCCTGGCACGG
UQCRC1-ChIP-R	ATGTTGCCCAGGCTGATCTTAAAC
**Synthetic interfering RNA**	
**Symbol**	**Sequence (5’- 3’)**
Scramble-sense	UUCUCCGAACGUGUCACGUTT
Scramble-antisense	ACGUGACACGUUCGGAGAATT
homo-ZHX2-1-sense	GCAGAACUGGAUCGGCUAATT
homo-ZHX2-1-antisense	UUAGCCGAUCCAGUUCUGCTT
homo-ZHX2-2-sense	CGAGGAGUCGAGCGUUGTGTT
homo-ZHX2-2- antisense	CACAACGCUCGACUCCUCGTT
homo-PGC-1α-1-sense	GUCGCAGUCACAACACUUATT
homo-PGC-1α-1-antisense	UAAGUGUUGUGACUGCGACTT
homo-PGC-1α-2-sense	GUGUGAUUUAUGUCGGUAATT
homo-PGC-1α-2-antisense	UUACCGACAUAAAUCACACTT
homo-FBXW7-sense	ACAGGACAGUGUUUACAAATT
homo-FBXW7-antisense	UUUGUAAACACUGUCCUGUTT
