# Supplementary material for: ZHX2 emerges as a negative regulator of mitochondrial oxidative phosphorylation during acute liver injury
Source: Nat Commun. 2023 Nov 18;14:7527. doi: 10.1038/s41467-023-43439-0 (PMC10657347; doi:10.1038/s41467-023-43439-0)
Supplement: Supplementary file 3 — Reporting Summary [file 41467_2023_43439_MOESM3_ESM.pdf]

nature portfolio

Corresponding author(s): Chunhong Ma and Xuetian Yue

Last updated by author(s): Oct 16, 2023

Reporting Summary

Nature Portfolio wishes to improve the reproducibility of the work that we publish. This form provides structure for consistency and transparency in reporting. For further information on Nature Portfolio policies, see our Editorial Policies and the Editorial Policy Checklist.

Statistics

For all statistical analyses, confirm that the following items are present in the figure legend, table legend, main text, or Methods section.

n/a	Confirmed	
☐	☒	The exact sample size (*n*) for each experimental group/condition, given as a discrete number and unit of measurement
☐	☒	A statement on whether measurements were taken from distinct samples or whether the same sample was measured repeatedly
☐	☒	The statistical test(s) used AND whether they are one- or two-sided<br>*Only common tests should be described solely by name; describe more complex techniques in the Methods section.*
☒	☐	A description of all covariates tested
☐	☒	A description of any assumptions or corrections, such as tests of normality and adjustment for multiple comparisons
☐	☒	A full description of the statistical parameters including central tendency (e.g. means) or other basic estimates (e.g. regression coefficient) AND variation (e.g. standard deviation) or associated estimates of uncertainty (e.g. confidence intervals)
☐	☒	For null hypothesis testing, the test statistic (e.g. *F*, *t*, *r*) with confidence intervals, effect sizes, degrees of freedom and *P* value noted<br>*Give P values as exact values whenever suitable.*
☒	☐	For Bayesian analysis, information on the choice of priors and Markov chain Monte Carlo settings
☒	☐	For hierarchical and complex designs, identification of the appropriate level for tests and full reporting of outcomes
☐	☒	Estimates of effect sizes (e.g. Cohen's *d*, Pearson's *r*), indicating how they were calculated

Our web collection on statistics for biologists contains articles on many of the points above.

Software and code

Policy information about availability of computer code

Data collection	Flow cytometry data were acquired with beckman CytoFLEX S connected with CytExpert (Software version 2.2).<br>RT-qPCR data were acquired using BioRad C1000 Thermal Cycler CFX96 Real-Time System with ChamQ Universal SYBR qPCR Master Mix (Vazyme Biotech, Q711).<br>The oxygen consumption rate (OCR) data were acquired with XF96 Seahorse Extracellular Flux Analyzer (Agilent,Seahorse XFe96).<br>Wester blots were imaged on Tanon 4600 using automatic exposure settings.<br>Immunostaining images were acquired by Olympus fluorescence microscope (DP80) and DeltaVision OMX SR super resolution imaging system (GE Healthcare).
Data analysis	Immunostaining and Western Blot quantification was done using ImageJ (version 1.51), and further analysis and calculations were done using Microsoft Excel and GraphPad Prism (version 8).<br>The oxygen consumption rate was done using Seahorse XF report generator wave(version2.6.1) and Datlab(version 7.4),<br>Analysis of qPCR results were done using Microsoft excel and GraphPad Prism (version 8).<br>All RNAseq data was analyzed using Sangerbox tools (version 1.0.8) and R studio (https:///rstudio.com/).

For manuscripts utilizing custom algorithms or software that are central to the research but not yet described in published literature, software must be made available to editors and reviewers. We strongly encourage code deposition in a community repository (e.g. GitHub). See the Nature Portfolio guidelines for submitting code & software for further information.

Data

Policy information about availability of data

All manuscripts must include a data availability statement. This statement should provide the following information, where applicable:

- Accession codes, unique identifiers, or web links for publicly available datasets
- A description of any restrictions on data availability
- For clinical datasets or third party data, please ensure that the statement adheres to our policy

The RNA-seq data in this publication have been deposited in the National Center for Biotechnology Information Sequence Read Archive and is accessible through accession PRJNA754419 (https://www.ncbi.nlm.nih.gov/sra/?term=PRJNA754419) and the ChIP-seq data in this publication have been deposited in the National Center for Biotechnology Information Sequence Read Archive and is accessible through accession PRJNA798889 (https://www.ncbi.nlm.nih.gov/sra/?term=PRJNA798889). The proteomics data have been deposited to the ProteomeXchange Consortium via the PRIDE partner repository with the dataset identifier PXD027897 (https://www.ebi.ac.uk/pride/archive/projects/PXD027897).
GSEA analysis were performed with publicly available data, including APAP-induced acute liver failure (ALF) (GSE74000).

Research involving human participants, their data, or biological material

Policy information about studies with human participants or human data. See also policy information about sex, gender (identity/presentation), and sexual orientation and race, ethnicity and racism.

Reporting on sex and gender	A total of 27 samples (16females and 11 males) from patients with drug-induce liver injury (DILI) were collected from Qilu Hospital of Shandong University and Beijing Ditan Hospital Capital Medical University. Subjects elegible for this study were of all sexes (aged 14-76).
Reporting on race, ethnicity, or other socially relevant groupings	Our samples were obtained from one populations, 18 females and 13 males,the age range of the cohort from which our samples came was 14 to 76 years old.
Population characteristics	The samples used in this study were from diagnosed drug-induce liver injury (aged 14-76).
Recruitment	The 27 samples were collected from Qilu Hospital of Shandong University and Beijing Ditan Hospital Capital Medical University . The authors do not see any potential bias in the generation or interpretation of the data reported in this study.
Ethics oversight	These studies were approved by the Research Ethics Committees of Qilu Hospital, Shandong University (KYLL-202209-033) and Beijing Ditan Hospital Capital Medical University (2021-041-01).

Note that full information on the approval of the study protocol must also be provided in the manuscript.

Field-specific reporting

Please select the one below that is the best fit for your research. If you are not sure, read the appropriate sections before making your selection.

☒ Life sciences ☐ Behavioural & social sciences ☐ Ecological, evolutionary & environmental sciences

For a reference copy of the document with all sections, see nature.com/documents/nr-reporting-summary-flat.pdf

Life sciences study design

All studies must disclose on these points even when the disclosure is negative.

Sample size	Sample sizes were chosen based on preliminary data demonstrating statistically significant differences for each specific assay
Data exclusions	No data was excluded.
Replication	All attempts at replication were successful. Findings were replicated in at least three biological independent samples each.
Randomization	For animal experiments, age-matched mice with different genotypes were randomly divided into different experimental groups. For cell culture experiments, cells with different genotypes or treatments were randomly divided into different experimental groups.
Blinding	Investigators were blinded during sample preparation and data collection, but not blinded during data analysis. Because information of group is required to perform comparison. Samples were analyzed with the same protocol by different investigators.

Reporting for specific materials, systems and methods

We require information from authors about some types of materials, experimental systems and methods used in many studies. Here, indicate whether each material, system or method listed is relevant to your study. If you are not sure if a list item applies to your research, read the appropriate section before selecting a response.

Materials & experimental systems

n/a	Involved in the study
	☒ Antibodies
	☒ Eukaryotic cell lines
☒	☐ Palaeontology and archaeology
	☒ Animals and other organisms
☒	☐ Clinical data
☒	☐ Dual use research of concern
☒	☐ Plants

Methods

n/a	Involved in the study
	☒ ChIP-seq
	☒ Flow cytometry
☒	☐ MRI-based neuroimaging

Antibodies

Antibodies used

Primary antibodies from Proteintech: anti-β-actin (66009-1-Ig, Clone: 2D4H5, 1:5,000), anti-ZHX2 (20136-1-AP, 1:4,000), anti-PCNA (10205-2-ap, 1:4,000), anti-CyclinA2 (18202-1-AP,1:2,000), anti-CyclinD1 (60186-1-Ig,1:2,000), anti-NRF1 (12482-1-AP,1:2,000), anti-TFAM (22586-1-AP,1:2,000), anti-PGC-1α (66369-1-Ig,1:2,000), anti-NDUFB9 (15572-1-AP,1:1,000), anti-COX7C (11411-2-AP,1:1,000), anti-SDHA (14865-1-AP,1:2,000), anti-UQCRC1 (21705-1-AP,1:2,000).
Primary antibodies from Abcam: anti-FBXW7 (ab109617,1:1,000), anti-Ki67 (ab15580,1:200), anti-Brdu (ab1893,1:200), anti-HA-ChIP Grade (ab9110,1:500).
Primary antibodies from Abclonal: anti-TOM20 (A19403,Clone:ARC5002-01,1:1,00).
Primary antibodies from MBL: anti-HA (M180-3,1:5000), anti-FLAG (M185-3,1:5000).
Secondary HRP-conjugated anti-mouse (Proteintech, SA00001-1, 1:5,000) or anti-rabbit IgG secondary antibodies (Proteintech, SA00001-2, 1:5,000).

Validation

All antibodies used in our study have been validated and detailed information could be found on the websites from manufactures as listed below:
anti-β-actin (66009-1-Ig, Clone: 2D4H5, 1:5,000):https://www.ptgcn.com/products/Pan-Actin-Antibody-66009-1-Ig.htm
anti-ZHX2 (20136-1-AP, 1:4,000):https://www.ptgcn.com/products/ZHX2-Antibody-20136-1-AP.htm
anti-PCNA (10205-2-ap, 1:4,000):https://www.ptgcn.com/products/PCNA-Antibody-10205-2-AP.htm
anti-CyclinA2 (18202-1-AP,1:2,000):https://www.ptgcn.com/products/CCNA2-Antibody-18202-1-AP.htm
anti-CyclinD1 (60186-1-Ig,1:2,000):https://www.ptgcn.com/products/CCND1-Antibody-60186-1-Ig.htm
anti-NRF1 (12482-1-AP,1:2,000):https://www.ptgcn.com/products/NRF1-Antibody-12482-1-AP.htm
anti-TFAM (22586-1-AP,1:2,000):https://www.ptgcn.com/products/TFAM-Antibody-22586-1-AP.htm
anti-PGC-1α (66369-1-Ig,1:2,000):https://www.ptgcn.com/products/PPARGC1A-Antibody-66369-1-Ig.htm
anti-NDUFB9 (15572-1-AP,1:1,000):https://www.ptgcn.com/products/NDUFB9-Antibody-15572-1-AP.htm
anti-COX7C (11411-2-AP,1:1,000):https://www.ptgcn.com/products/COX7C-Antibody-11411-2-AP.htm
anti-SDHA (14865-1-AP,1:2,000): https://www.ptgcn.com/products/SDHA-Antibody-14865-1-AP.htm
anti-UQCRC1 (21705-1-AP,1:2,000):https://www.ptgcn.com/products/UQCRC1-Antibody-21705-1-AP.htm
anti-FBXW7 (ab109617,1:1,000):https://www.abcam.cn/products/primary-antibodies/fbxw7-antibody-ab109617.html
anti-Ki67 (ab15580,1:200):https://www.abcam.cn/products/primary-antibodies/ki67-antibody-ab15580.html
anti-Brdu (ab1893,1:200):https://www.abcam.cn/products/primary-antibodies/brdu-antibody-proliferation-marker-ab1893.html
anti-HA-ChIP Grade (ab9110,1:500):https://www.abcam.cn/products/primary-antibodies/ha-tag-antibody-chip-grade-ab9110.html
anti-TOM20 (A19403,Clone:ARC5002-01,1:1,00):https://abclonal.com.cn/catalog/A19403
anti-HA (M180-3,1:5000):https://www.mbl-chinawide.cn/search012?keyword=M180-3&field=class-39&order=&limit=100
anti-FLAG (M185-3,1:5000):https://www.mbl-chinawide.cn/search012?keyword=M185-3L
Secondary HRP-conjugated anti-mouse (Proteintech, :https://www.ptgcn.com/products/HRP-conjugated-Affinipure-Goat-Anti-Mouse-IgG-H-L-secondary-antibody.htm, 1:5,000)
anti-rabbit IgG secondary antibodies (Proteintech, SA00001-2, 1:5,000):https://www.ptgcn.com/products/HRP-conjugated-Affinipure-Goat-Anti-Rabbit-IgG-H-L-secondary-antibody.htm

Eukaryotic cell lines

Policy information about cell lines and Sex and Gender in Research

Cell line source(s)

Huh7(SCSP-526), HepG2(SCSP-510) and HEK293T(SCSP-502) cells were purchased from Shanghai Institute of Cell Biology.

Authentication

All cell lines used in this study were authenticated by the supplier. Cell authentication is based on their morphology, growth conditions and specific gene expression.

Mycoplasma contamination

All cell lines were tested negative for mycoplasma contamination.

Commonly misidentified lines (See ICLAC register)	No commonly misidentified lines were used in this study.

Animals and other research organisms

Policy information about studies involving animals; ARRIVE guidelines recommended for reporting animal research, and Sex and Gender in Research

Laboratory animals	Albumin-Cre mice were obtained from Jackson Laboratory. Zhx2 floxed mice were gifted by Prof. Brett T Spear from University of Kentucky. Hepatocyte-specific Zhx2 deficient mice (Zhx2-KOhep) were generated by crossing Albumin-Cre and Zhx2 floxed mice. Their littermates without Albumin-Cre were defined as Zhx2-WT mice. C57BL/6 mice (6-8 weeks of age) were purchased from GemPharmatech LLC. All mice were maintained under specific pathogen-free conditions with a 12-h light, 12-h dark cycle and given free access to food and water.
Wild animals	The study did not include wild animals.
Reporting on sex	There is no gender difference for the ZHX2 effect of liver regeneration.We have added this information in Material and Methods section.
Field-collected samples	The study did not include field-collected samples.
Ethics oversight	The study was approved by the Ethics Committee of School of Basic Medical Sciences, Shandong University. Animal Ethics Number: ECSBMSSDU2018-1-031.

Note that full information on the approval of the study protocol must also be provided in the manuscript.

Plants

Seed stocks	n/a
Novel plant genotypes	n/a
Authentication	n/a

ChIP-seq

Data deposition

- [x] Confirm that both raw and final processed data have been deposited in a public database such as GEO.
- [x] Confirm that you have deposited or provided access to graph files (e.g. BED files) for the called peaks.

Data access links *May remain private before publication.*	Data in this study is publicly available in the National Center for Biotechnology Information Sequence Read Archive (PRJNA798889).
Files in database submission	FASTQ files for Input and IP ChIP-seq
Genome browser session (e.g. UCSC)	No applicable-visualized data using IGV

Methodology

Replicates	ChIP-seq was performed with one biological replicate for each condition
Sequencing depth	Input:clean reads 17271897. IP:clean reads 19606094
Antibodies	Abcam,anti-HA (ab9110)
Peak calling parameters	After mapping reads to the reference genome, we used the MACS2 (version 2.1.0) peak calling software to identify regions of IP enrichment over background. A q-value threshold of 0.05 was used for all data sets. After peak calling, the distribution of chromosome distribution, peak width, fold enrichment, significant level and peak summit number per peak were all displayed.
Data quality	Raw data (raw reads) of fastq format were firstly processed using fastp (version 0.19.11) software. In this step, clean data (clean reads) were obtained by removing reads containing adapter, reads containing ploy-N and low quality reads from raw data. At the same time, Q20, Q30 and GC content of the clean data were calculated. All the downstream analyses were based on the clean data with high quality.
Software	R(version 3.6),IGV software (version 2.7.2)

Flow Cytometry

Plots

Confirm that:

- [x] The axis labels state the marker and fluorochrome used (e.g. CD4-FITC).
- [x] The axis scales are clearly visible. Include numbers along axes only for bottom left plot of group (a 'group' is an analysis of identical markers).
- [x] All plots are contour plots with outliers or pseudocolor plots.
- [x] A numerical value for number of cells or percentage (with statistics) is provided.

Methodology

Sample preparation	Primary hepatocyte and Huh7 cells were incubated with 1 µM MitoTracker deep Red FM probe (Thermo Fisher Scientific, M22426), 2 µM MitoProbe™ JC-1(Thermo Fisher Scientific, M34152) or 20 µM MitoProbe™ TMRM (Thermo Fisher Scientific, M20036) at 37 °C for 30 min, respectively. After staining was completed, the cells were gently washed three times with warm PBS, and detached to a single cell suspension.
Instrument	CytoFLEX S flow cytometer (Beckman)
Software	CytExpert, FlowJo software (version 8.5.2)
Cell population abundance	Cell population abundances were analyzed using FlowJo software and reported in figures. This study did not involve any cell-sorting experiments.
Gating strategy	We used standard gating strategies:<br>1. Gating on the typical leukocyte population based on FSC-SSC signals<br>2. Single cells were selected using FSC-W, FSC-A<br>3. Cell proportion and staining intensity were identified based on specific probe markers

- [ ] Tick this box to confirm that a figure exemplifying the gating strategy is provided in the Supplementary Information.
